# Type IV Sacrococcygeal Teratoma Displacing the Urinary Bladder: Unique Magnetic Resonance Imaging

**DOI:** 10.1155/2016/1423157

**Published:** 2016-06-20

**Authors:** Sahar Eftekharzadeh, Sorena Keihani, Mehdi Fareghi, Alireza Alamsahebpour, Abdol-Mohammad Kajbafzadeh

**Affiliations:** Pediatric Urology Research Center, Pediatric Center of Excellence, Tehran University of Medical Sciences, Tehran 1419433151, Iran

## Abstract

Type IV sacrococcygeal teratoma is a rare pediatric tumor that is confined to the presacral area with no external component. The signs and symptoms often arise due to mass effect and compression of adjacent organs. Urinary retention is an uncommon presenting symptom in these patients. A wide spectrum of imaging findings may be encountered in cases with sacrococcygeal teratoma because of variability of tumor size and components. We hereby present a unique magnetic resonance urography finding in a type IV sacrococcygeal teratoma which caused bladder displacement. A meticulous and complete resection of tumor with special attention to the pelvic plexus led to preservation of normal voiding function and normal bowel function in this patient.

## 1. Introduction

Sacrococcygeal teratoma (SCT), though the most common germ cell tumor in children, is a rare entity occurring in 1 in every 35000 to 40000 births [1]. SCTs are classified into four types according to the anatomical extent of the tumor with type 1 being mostly external and mostly compromising postsacral area and type IV being internally confined to the presacral area [2].

Different imaging techniques play an important role in diagnosis and evaluation of SCTs, especially in type IV Altman SCTs that are not visualized externally. However, a wide spectrum of imaging findings may be encountered, given the wide variety in these tumors and different solid or cystic components. We hereby present unique magnetic resonance imaging (MRI) and magnetic resonance urography (MRU) findings in a boy with type IV SCT and we also briefly discuss the management of this patient.

## 2. Case Presentation

A 32-month-old boy presented to our pediatric urology center with multiple episodes of urinary retention in the previous month. He was otherwise healthy and did not have any history of previous surgical or medical conditions. Physical examination revealed a large nontender mass in the lower abdomen. Routine laboratory data were all within normal limits. Plain radiography of pelvis showed a mass composed of soft tissue and calcifications in its lower part, as well as a short sacrum. Ultrasonography (US) revealed a pelvic mass with both cystic and solid components and also bilateral hydronephrosis.

**Figure 1:**
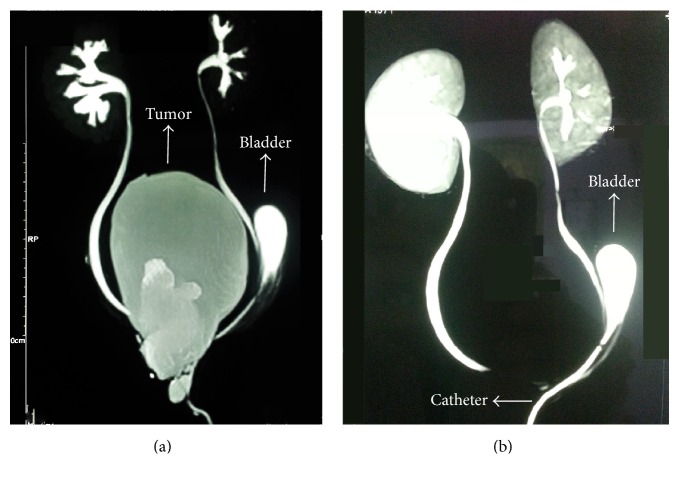
(a) Static magnetic resonance urogram showing the tumor displacing the bladder. (b) Functional magnetic resonance imaging showing ureters, displaced bladder, and hydronephrosis in both kidneys.

Computed tomography (CT) was taken in the district hospital before patient's admission to our center. It confirmed previous findings but it did not delineate the exact relationship of the mass with spinal column to narrow down the differential diagnoses. MRI and MRU were performed to investigate the characteristics of the mass and its possible connection to the spinal cord. MRI showed a 144 × 63 mm^2^ large presacral mass with septated cystic and solid components arising from coccygeal area without any connections to spinal canal. The lesion caused complete displacement of the bladder anteriorly and to the left side. The right ureter had an S-shaped course around the inferior portion of the mass to enter the shifted bladder (Figure 1). Voiding cystography also confirmed bladder dislocation to the left lower part of the abdomen and did not show any vesicoureteral reflux.

**Figure 2:**
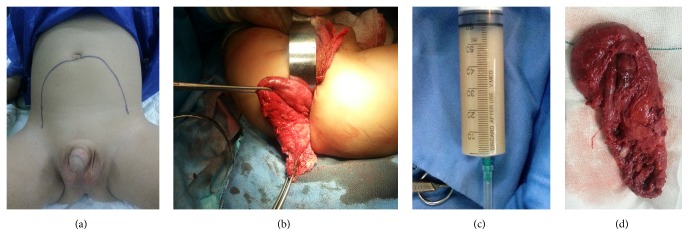
(a) Tumor extension. (b) Tumor resection through the posterior sagittal incision. (c) Turbid fluid withdrawn from the cystic component of tumor. (d) Gross appearance of the tumor.

Surgical resection of the tumor was recommended. Prior to incision, a urinary catheter and a rectal tube were fixed to guide the surgeon. First, in prone (jack-knife) position a posterior sagittal incision was made. With guidance of rectal tube the mass was carefully separated from rectum, but due to its large volume complete excision through posterior sagittal incision was not possible. Subsequently, the patient's position was changed to supine and an anterior midline incision was made to access the tumor from abdomen and to complete the excision (Figure 2(b)). Components of the large cystic mass, including a turbid fluid (Figure 2(c)) and hairs, were carefully evacuated by suction to avoid tumor rupture and spillage of components in the surgical field. Tumor adhesion to different organs especially the sacrum was finally released and the tumor was resected completely (Figure 2(d)). Meticulous approach was used without using unipolar electrocauter to avoid damage to sacral plexus.

Histopathological investigations of the mass showed a cystic neoplasm composed of various mature tissue components including skin, pilosebaceous glands, well-developed respiratory and gastric epithelium, and glial tissue with numerous psammoma bodies. Based on pathological studies the final diagnosis was confirmed as mature type IV SCT.

## 3. Discussion

Type IV SCTs are the least common type in children and may cause diagnostic difficulties since they are not visualized externally and the diagnosis is largely dependent on imaging studies. These tumors are usually asymptomatic but if symptoms arise, they are mainly caused by tumor's mass effect on adjacent organs. This can lead to bowel and bladder dysfunction (e.g., constipation, obstruction, and incontinence), venous or lymphatic obstruction, or even lower extremity paralysis [3, 4]. In some cases SCTs present with urological symptoms; however, urinary retention is a rare and unusual presentation [5, 6]. The large pelvic mass in our patient caused bladder displacement and urinary retention as the presenting symptom without any associated symptoms. This also led to a unique MRU finding of the cystic mass replacing the bladder in the pelvic area (Figure 1).

Risk of recurrence might be high especially in immature and malignant SCTs and in case of incomplete resection of tumor, nonresection of coccyx, and spillage of tumor components during surgery [6]. The surgeon should properly drain the cystic lesion and avoid tumor rupture and spillage to reduce this risk [7]. Postsurgical urologic and gastrointestinal sequels including high grade reflux and bladder dysfunction are common in these patients due to mass effect of tumor or most likely because of intraoperative damage to sacral nerve plexus during extensive tumor resection. The urologic comorbidities are usually associated with more pelvic involvement; in other words, higher Altman grades are associated with further urologic sequela [3, 6, 8]. However, careful and precise resection with preservation of neural structures that supply bladder and anal sphincter may reduce this risk. Preoperative and postoperative urologic assessments, for instance urodynamic studies, are beneficial in recognition of neurourological dysfunctions of lower urinary tract [8, 9]. With this approach, our patient had normal voiding and ultrasound findings during the 18-month follow-up with preserved bowel and bladder function and continence.

We assume that magnetic resonance imaging is an excellent modality for precise anatomical diagnosis and surgical planning in SCTs. The imaging findings in SCTs are widely variable based on tumor size, location, and constitution. Careful and complete tumor resection with special attention to neural structures is needed to reduce risk of recurrence as well as functional urologic sequels.
